# Evaluation of Phytosomal Curcumin as an Anti-inflammatory Agent for Chronic Glial Activation in the GFAP-IL6 Mouse Model

**DOI:** 10.3389/fnins.2020.00170

**Published:** 2020-03-12

**Authors:** Faheem Ullah, Huazheng Liang, Garry Niedermayer, Gerald Münch, Erika Gyengesi

**Affiliations:** ^1^Department of Pharmacology, School of Medicine, Western Sydney University, Campbelltown, NSW, Australia; ^2^Department of Neurology, Translational Research Institute of Brain and Brain-like Intelligence, Shanghai Fourth People’s Hospital Affiliated to Tongji University School of Medicine, Shanghai, China; ^3^School of Science, Western Sydney University, Campbelltown, NSW, Australia; ^4^NICM Health Research Institute, Western Sydney University, Campbelltown, NSW, Australia

**Keywords:** microglia activation, astrocytes, Iba-1, TSPO, GFAP, neuroinflammation, stereology

## Abstract

Chronic glial activation is characterized by an increased number of activated microglia and astroglia; these secrete free radicals and cytotoxic cytokines, subsequently causing neuronal damage. This study investigated the hypothesis that a soy-lecithin based phytosomal curcumin formulation can decrease glial activation in the brains of GFAP-IL6 mice, a model of chronic glial activation, which exhibits gliosis in various regions of the brain. Three doses of Meriva curcumin (MC) (874, 436, and 218 PPM) were fed to 3-month-old GFAP-IL6 and wild-type (WT) mice for 4 weeks. As markers of glial activation, the total numbers of Iba-1^+^ and TSPO^+^ microglia and macrophages, and GFAP^+^ astrocytes, were determined in the cerebellum and hippocampus by immunohistochemistry and unbiased stereology. Furthermore, the morphology of the glial cells was assessed by confocal microscopy and Sholl analysis. Administration of phytosomal curcumin led to a dose-dependent reduction in neuroinflammatory markers. Phytosomal curcumin (874 PPM) decreased the number of microglia by 26.2% in the hippocampus and by 48% in the cerebellum of the GFAP-IL6 mice compared with the GFAP-IL6 mice on normal food. Additionally, GFAP^+^ astrocyte numbers in the hippocampus of the GFAP-IL6 mice were decreased by 42%. The GFAP-IL6 mice exhibited a different microglial morphology to the WT mice, showing an increased soma size and perimeter. This difference was significantly reduced by the 874 PPM phytosomal curcumin dose. Our findings demonstrate that phytosomal curcumin is able to attenuate the inflammatory pathology, and potentially reverse the detrimental effects of chronic glial activation.

## Introduction

Neuroinflammation is a defense response of the central nervous system (CNS) to injury, infection, or the presence of toxic metabolites. Acute neuroinflammation is a self-protective reaction aimed at eliminating harmful stimuli and restoring tissue integrity. However, if neuroinflammation becomes chronic, it can be harmful. Chronic neuroinflammation is both a feature and a potential cause of many neurodegenerative diseases such as Alzheimer’s disease and dementia (Arends et al., 2000). Interleukin 6 (IL-6) is a pleiotropic, regulatory cytokine that may play a pathogenic role in dysregulating inflammatory responses (Akira et al., 1990). Microglia and astrocytes play a key role in the CNS’s innate immunity. The major function of microglia is to clear damaged neurons and foreign pathogens, while astrocytes help to remove debris and toxins from the cerebrospinal fluid. When activated, microglia and astrocytes produce a variety of proinflammatory cytokines and neurotoxic factors, such as reactive oxidative species (ROS), leading to neuronal damage, neuroinflammation (Wang et al., 2015; Tjalkens et al., 2017), and subsequent cognitive deficits (Rosenblat et al., 2014; Heneka et al., 2015; Kirkley et al., 2017). Microglia are the key neuroinflammatory cells as they are activated in response to brain inflammation and release the ROS and cytokines that cause neurotoxicity (Beggs and Salter, 2007; Block et al., 2007). TSPO is a translocator protein expressed in the nervous system, specifically on the outer mitochondrial membrane (Chen and Guilarte, 2008). It is predominantly expressed in the microglia, astrocytes, and macrophages in the blood vessels (Kuhlmann and Guilarte, 2000; Wilms et al., 2003). TSPO represents a novel macrophage marker, as neurological insults and chronic neuroinflammation induce TSPO expression in the nervous system (Kuhlmann and Guilarte, 2000; Casellas et al., 2002). Astrocytes are cells that are vital to the CNS, playing an important role in coupling neuronal organization to blood flow and maintaining, regulating, and altering neuronal synaptic junctions (Zhang and Haydon, 2005). Astrocytes are highly reactive cells that respond to adverse neural insults, such as neuroinflammation, trauma and neurodegeneration (Sofroniew, 2009). Previous studies conducted on GFAP-IL6 mice have shown a larger number of GFAP^+^ astrocytes in both the cerebellum and cortical areas compared with wild type (WT) mice (Chiang et al., 1994).

Morphological features are one of the important characteristics of microglial cells. Under normal circumstances, resting microglia with a ramified morphology continuously scan the nervous system (Nimmerjahn et al., 2005). During inflammatory conditions, the microglial cells have been observed to go into an activated state, where they become amoeboid-like in their morphology, becoming de-ramified microglia, which are characterized by a swollen cell body and thick processes. Similarly, astrocytic morphological changes are a hallmark of neurodegenerative disease; in certain physiological conditions, astrocytes have a bushy or spongiform shape, with fine, delicate processes emerging from the soma. During neuroinflammation, when astrocytes are activated, they are characterized by an increase in size, number and thickness of processes, and an increased level of GFAP expression (Wilhelmsson et al., 2006).

Curcumin is an active component of turmeric, constituting 2–5% of the spice (Aggarwal et al., 2003). It has low toxicity and high preclinical efficacy, which makes it a good natural drug candidate against inflammatory conditions (Burgos-Moron et al., 2010; Ullah et al., 2017). It is a potent cytokine-suppressive anti-inflammatory drug (CSAID) and exerts a broad range of anti-inflammatory effects. Curcumin downregulates the expression of cyclooxygenase-2 (COX-2), inducible nitric oxide synthase (iNOS), tumor necrosis factor (TNF-α), IL-1, -2, -6, -8, and -12 (Abe et al., 1999; Hansen et al., 2010). Initially, results of clinical trials conducted with curcumin were disappointing, probably due to the poor bioavailability and rapid metabolism of the compound (Jurenka, 2009). In order to improve the bioavailability of curcumin, a variety of curcumin formulations were developed, including phytosomal Meriva curcumin (MC), produced by Indena S.p.A, Italy, which consists of curcumin combined with soy-lecithin (Antiga et al., 2015).

GFAP-IL6 mice have been used in this study as a model of chronic glial cell activation. In the GFAP-IL6 mouse model, the murine IL-6 gene is expressed by astrocytes under the transcriptional control of the glial fibrillary acidic protein (GFAP) promoter, resulting in brain-specific overexpression of IL6 and chronic low-grade neuroinflammation, characterized by microglia and astrocyte activation throughout the lifespan (Gyengesi et al., 2019). This has been demonstrated via a significant increase in Iba-1^+^ and TSPO^+^ microglia and macrophages, GFAP^+^ astroglia and neurodegeneration predominantly in the cerebellum (Campbell et al., 1993), leading to the activation of microglia (microgliosis) and astrocytes (astrocytosis) (Gyengesi et al., 2018) and a range of structural and functional neurological impairments that typify various neurodegenerative diseases (Campbell et al., 1993). Previous studies have reported that GFAP-IL6 transgenic mice display a high level of IL6 expression in the cerebellum compared with other brain regions (Campbell et al., 1993; Quintana et al., 2009). A recent study conducted by our lab in the same animal model has confirmed that the number of Iba-1^+^ cells in GFAP-IL6 mice is significantly larger compared with that of the WT mice (Gyengesi et al., 2018). This paper represents the first study to evaluate the effect of MC in the GFAP-IL6 mouse model. MC could be a potential suppressive agent against chronic neuroinflammation through the modulation of astroglial and microglial activity. To determine how MC affects the number and morphology of activated (Iba-1^+^, TSPO^+^) microglia and activated (GFAP^+^) astroglia in the brain, stereological counting and three-dimensional (3D) reconstruction techniques were applied to assess reactive and non-reactive microglia and astrocytes in the hippocampus and the cerebellum in an unbiased manner.

## Materials and Methods

### Animals

Wild-type (C57BL/6) and GFAP-IL6 mice of mixed genders weighing 20–30 g were housed in the animal facility of the School of Medicine, Western Sydney University under a temperature-controlled environment, with a normal 12 h/12 h light–dark cycle at 23°C and 60 ± 10% humidity, and were provided with food and water *ad libitum*. The experimental procedures were approved by the Western Sydney University Animal Care and Ethics Committee (approval ID: A11393) and carried out in accordance with the rules established by the National Health and Medical Research Council of Australia. Three-month-old heterozygous GFAP-IL6 mice and their non-transgenic littermates (WT C57BL/6) were used.

### Grouping of Animals and Feeding With Phytosomal Curcumin Containing Food Pellets

Animals were randomly assigned to five groups: WT C57BL/6 mice fed with control food pellets, GFAP-IL6 mice fed with control food pellets and three groups of GFAP-IL6 mice fed with food pellets containing 874, 436, and 218 PPM of MC (Table 1 and Supplementary Table S1). Meriva curcumin (MC) was supplied by Indena S.p.A, Italy, and incorporated into the mouse chow (Specialty Feeds, Perth, Australia). MC was mixed with powdered mouse food, and around 15% water was added before introduced the mixed diet to the pellet machine. In the pelleting machine, the temperature of the product would not exceed 43C. After pelleting the moisture content had to be reduced to stabilize the diet against microbial storage damage. This was done by loading the pellets into a shallow tray in an air dryer set at 65C for 3 h. Over the drying cycle the temperature of the pellets was increased slowly from 25C to 45C. The mouse chow pellets were stored at room temperature in vacuum packed bags in the animal housing facility at the School of Medicine, Western Sydney University. At the age of 3 months, the mice were fed for 4 weeks with normal food or MC. The cage food trays were monitored weekly to record food consumption. At the age of 4 months, the animals were perfused for histology.

**TABLE 1:** Number of mice per cohorts.

Cohort genotype	Type of food	Number of mice/male/female (M/F) used		
		Iba-1	GFAP	TSPO
WT	Normal food	6 (3M, 3F)	4 (2M, 2F)	6 (3M, 3F)
GFAP-IL6	Normal food	7 (4M, 3F)	4 (2M, 2F)	5 (4M, 1F)
GFAP-IL6	218 PPM MC	7 (3M, 4F)	5 (1M, 4F)	6 (2M, 4F)
GFAP-IL6	436 PPM MC	6 (3M, 3F)	6 (3M, 3F)	5 (2M, 3F)
GFAP-IL6	874 PPM MC	7 (3M, 4F)	7 (3M, 4F)	7 (3M, 4F)

### Histology and Tissue Sample Preparation

For histological analysis, the tissue samples were prepared from all the experimental cohorts. The mice were anesthetized with pentobarbitone (30–50 mg/kg i.p.) and were transcardially perfused with 30 ml of 0.9% normal saline using a peristaltic pump, followed by 60 ml of 4% cold paraformaldehyde (Merck) (in 0.1 M phosphate buffer). The brains were harvested and post-fixed in 4% paraformaldehyde for 24 h at 4°C, and then transferred to 30% sucrose (in 0.1 M PB solution) for cryoprotection. After the brains sank to the bottom of the container, they were embedded and frozen with 6% gelatin. Forty μm thick coronal sections were cut in eight series using a Leica CM 1950 cryostat.

### Immunohistochemistry

For bright field microscopy, immunohistochemistry assays were performed on every eighth section from the brains to identify the microglial activation using microglia markers (Iba-1 and TSPO). All washing and incubation procedures were performed using 0.1 M PBS unless stated otherwise. The sections were washed three times and treated with 1% H2O2 before being incubated for 2 h in the blocking solution (2% goat serum) to block the non-specific antigen-binding sites. They were then incubated in the primary rabbit-anti-Iba1 antibody (1:500, Wako, # 019-19741) and rabbit anti TSPO (1:500, Merck, #ABC139) solutions for 2 days at 4°C and, subsequently, in the secondary antibody (1:200, biotinylated goat anti-rabbit IgG; Life Technologies, #656140) for 2 h. The sections were washed three times and ABC solution (1:250, Vector Laboratories, # PK-6100) was applied for 2 h. The sections were then incubated in the developer solution containing 0.4 mg/ml DAB and 0.0006% hydrogen peroxide until an optimal color developed. Then, the sections were washed, mounted, dehydrated, coverslipped, and their images were captured with a Zeiss microscope (MBF Bioscience).

### Fluorescence Microscopy

Immunofluorescent staining was performed on every eighth section from the brains to identify the astroglial activation using GFAP as an astroglial marker. The sections were washed with 0.1 M PBS in order to remove the gelatin and were incubated in the primary rabbit-anti-GFAP antibody (1:500, Dako, #20023331) solution for 1 day at 4°C, followed by fluorescence secondary antibody (1:200, goat anti-rabbit IgG, Alexa 488; Thermo Fisher Scientific, #1853312) for 2 h. The sections were washed, mounted, and coverslipped with a fluorescent mounting medium (Vector Laboratories, #H1400).

### Stereological Counting

The estimated number of glial cells in both the cerebellum and the hippocampus was counted on Iba1, TSPO and GFAP stained sections using the Zeiss AxioImager M2 microscope equipped with the MBF Biosciences StereoInvestigator (Bastide et al., 2014). The contour of the cerebellum and the hippocampus was first drawn under the 2.5x objective. The size of the counting frame for the WT mice was 130 × 130 μm for the cerebellum and 120 × 120 μm for the hippocampus. The size of the counting frame for the GFAP-IL6 mice was 60 × 60 μm for both the cerebellum and hippocampus. The counting grid was 1500 × 1000 μm for the cerebellum, and 800 × 800 μm for the hippocampus, for all of the cohorts. The guard zone was 1 μm at the top and the bottom of the sections. Microglia and astrocytes were plotted on the screen using a marker as the focus moved from the top to the bottom of the sections using a 63x oil objective. This led to a Gunderson coefficient error of less than 0.1 in all cases (*m* = 1). Due to technical limitations, it was not possible to discriminate between individual astrocytes in the cerebellum. Therefore, for this time point, only the representative pictures are shown. However, the number of GFAP^+^ cells in the hippocampus was measured under the above-mentioned parameters.

### Three-Dimensional Reconstruction of Astrocytes and Microglia

Samples were prepared as described above and the images were taken using a Confocal ZEISS Laser Scanning Microscope (LSM-5) with an argon laser and processed using the Zen 2009 software package. The Z-stacks were captured using a 20x objective and NA1.0 for reflective imaging, at a step size of 0.1 μm (unless otherwise specified). Reflective imaging was achieved using the 488 nm wavelength. For the 3D reconstruction of both astrocytes and microglia, Neurolucida 360 (MBF Bioscience) software was used. To better identify the objects and provide greater accuracy, all the images were taken using a 20x high power objective with the laser scanning confocal microscope. A total of 16–20 cells in the hippocampus and the cerebellum of the brain were traced in each experimental cohort and fully or partially reconstructed (only soma). After importing the Z-stacks to Neurolucida 360 software, the astrocytes and microglia were manually reconstructed along the required planes, obtaining a 3D image of each cell.

### Analysis of Reconstructed Cells

Morphometric data of each astrocyte and microglia were extracted by the software and thus, each reconstructed cell was subject to multiple parameters. The soma area, soma perimeter, convex two-dimensional (2D) area, convex perimeter, the total length of all processes, the total volume of all processes, the total density and the number of nodes (Branch points) and dendrites of all the processes were measured. In order to determine changes in the size of the cells in relation to the distance from the cell soma, Sholl analysis was performed for each microglial and astroglial cell (Bento-Torres et al., 2017). The Z-stack images of the live microglia and astrocytes were condensed into a maximum intensity and applied to the Sholl analysis in Neurolucida 360 software. Concentric circles (radii) originating from the soma were spaced 5 μm apart in this case. This analysis determined the number of intersections, the process length (μm), the surface area of the cells (μm2), the process volume (μm3), the process diameter (μm) and a number of nodes of the cells for each radius.

### Data Analysis

The estimated number of microglia and astrocytes in both the cerebellum and the hippocampus were compared between groups using one-way ANOVA in GraphPad Prism 6. The results were presented as mean ± SEM. Significance was indicated when p was less than 0.05. The distinct morphological features of microglia and astrocytes in the brain were compared between groups using a one-way ANOVA with Tukey’s post-test (^∗^*p* < 0.05, ^∗∗^*p* < 0.001, ^∗∗∗^*p* < 0.0001, mean ± SEM). Correlations of the convex hull area with the distinct morphological features of the microglia and astrocytes were analyzed using linear regression (R2) and correlation (^∗^*p* < 0.05) tests in GraphPad Prism 7. The ANCOVA test was used to compare slope differences in each specific morphological feature (^∗^*p* < 0.05) between the different experimental cohorts.

## Results

### MC Decreased the Number of Iba-1^+^ Microglia in the Hippocampus and the Cerebellum of GFAP-IL6 Mice

Phenotype and feed effects on the number of Iba-1^+^ microglial cells were determined. Immunohistochemistry and stereological counting of Iba-1^+^ microglia were performed in the hippocampus and cerebellum of GFAP-IL6 mice fed with normal food or increasing doses of MC.

**FIGURE 1:**
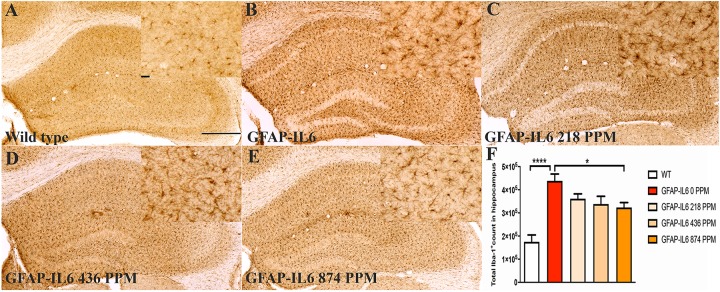
MC decreased the number of Iba-1^+^ microglia in the hippocampus of GFAP-IL6 mice. **(A–E)** Representative photomicrographs of immunohistochemical staining for the microglia (Iba-1^+^ cells) of the hippocampus. (Magnification 10x objective field, scale bar = 500 μm and 100 μm in inserts). Images are representative of at least six animals per group (*n* = 6). **(F)** Graphical representation of total Iba-1+ count in the hippocampus. The graph represents the mean ± SEM and significant differences were determined using one-way analysis of variance (ANOVA). Significance = ****p < 0.0001, *p < 0.05.

The hippocampus of the GFAP-IL6 mice fed with normal food had 438,550 ± 29,717 Iba-1^+^ microglia, which was significantly higher than that of the WT mice (175,192 ± 12,098) (*p* < 0.0001) (Figures 1A,B,F). In the MC fed GFAP-IL6 mice, a reduction in the number of Iba-1^+^ microglia was observed. The low doses, 218 PPM and 436 PPM, of MC reduced the number by 17.5% (361,644 ± 20,593) and 22% (338,483 ± 33,212) (Figures 1C,D,F), whereas 874 PPM of MC significantly reduced the number of microglia by 26.2% (323,451 ± 21,520) compared with the GFAP-IL6 normal food fed cohort (Figures 1E,F).

**FIGURE 2:**
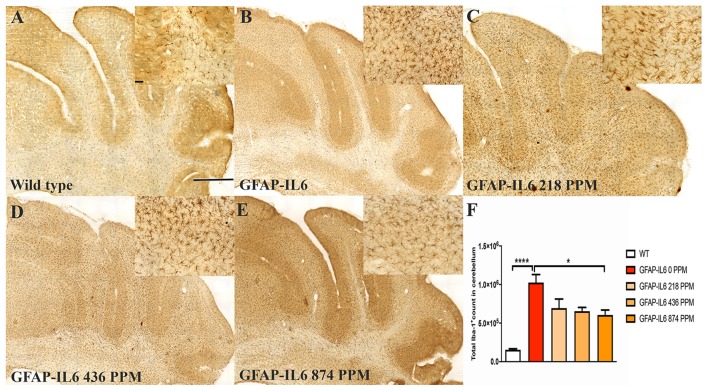
MC decreased the number of Iba-1^+^ microglia in the cerebellum of GFAP-IL6 mice. **(A–E)** Representative photomicrographs of immunohistochemical staining for the microglia (Iba-1^+^ cells) of the cerebellum. (Magnification 10x objective field, scale bar = 500μm and 100 μm in inserts). Images are representative of at least six animals per group (*n* = 6). **(F)** Graphical representation of total Iba-1^+^ count in the cerebellum. The graph represents the mean ± SEM and significant differences were determined using one-way analysis of variance (ANOVA). Significance = ****p < 0.0001, *p < 0.05.

In the cerebellum, the GFAP-IL6 mice had 1,025,205 ± 104,467 Iba-1^+^ microglia; this was significantly higher than that of the WT mice (154,058 ± 12,871) (*p* < 0.0001) (Figures 2A,B,F). A reduction in a total number of microglial cells was observed in all the MC dose groups compared with the normal food fed GFAP-IL6 group. The GFAP-IL6 mice fed with 218 PPM, 436 PPM and 874 PPM downregulated the Iba-1^+^ microglia by 32% (693,938 ± 117,878), 36% (654,996 ± 48,489) and 40% (605,085 ± 63,857), respectively (Figures 2C–F).

### MC Decreased the Number of TSPO^+^ Microglial Cells/and Macrophages in the Hippocampus and Cerebellum of the GFAP-IL6 Mice

The mitochondrial translocator protein (TSPO) is expressed in both macrophages and a portion of the microglia in the brain and has been shown to be upregulated in reactive microglia during inflammation (Cosenza-Nashat et al., 2009; Mirzaei et al., 2016). In addition to Iba-1, immunostaining was performed for TSPO as a microglial marker. Stereological quantification was performed in order to test whether there was a difference in TSPO^+^ microglia/macrophages between the WT and GFAP-IL6 mice, and whether MC changes the number of TSPO^+^ cells in the brains of the GFAP-IL6 mice.

**FIGURE 3:**
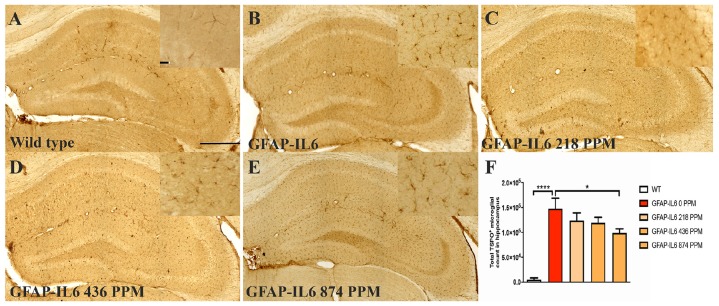
MC decreased the number of TSPO^+^ microglial cells/macrophages in the hippocampus of GFAP-IL6 mice. **(A–E)** Representative photomicrographs of immunohistochemistry staining for the microglia (TSPO^+^ cells) of the hippocampus. (Magnification 10x objective field, scale bar = 500 μm and 100 μm in inserts). Images are representative of at least six animals per group (*n* = 6). **(F)** Graphical representation of total TSPO^+^ microglial/macrophages count in the hippocampus. The graph represents the mean ± SEM and significant differences were determined using one-way analysis of variance (ANOVA). Significance = *p < 0.01, ****p < 0.0001.

In the hippocampus, the GFAP-IL6 mice had 147,803 ± 46,723 TSPO^+^ microglia/macrophages compared with the WT mice (4747 ± 3866) (*p* < 0.0001) (Figures 3A,B,F). The 218 PPM and 436 PPM doses of MC reduced the number of TSPO^+^ microglia/macrophages by 16% (123,769 ± 6266) and 19% (119,213 ± 11,229), respectively. The 874 PPM dose of MC significantly decreased the number by 33% (98,941 ± 21,413) (Figures 3C–F).

**FIGURE 4:**
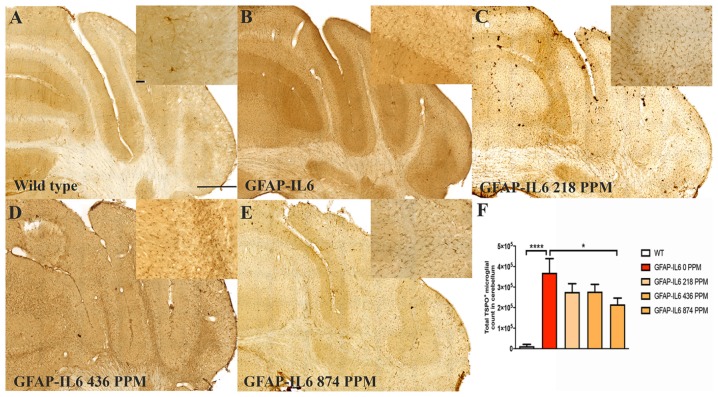
MC decreased the number of TSPO^+^ microglial cells/macrophages in the cerebellum of GFAP-IL6 mice. **(A–E)** Representative photomicrographs of immunohistochemistry staining for the microglia (TSPO^+^ cells) of the cerebellum. (Magnification 10x objective field, scale bar = 500μm and 100 μm in inserts). Images are representative of at least six animals per group (*n* = 6). **(F)** Graphical representation of total TSPO^+^ microglial/macrophages count in the cerebellum. The graph represents the mean ± SEM and significant differences were determined using one-way analysis of variance (ANOVA). Significance = *p < 0.01, ****p < 0.0001.

In the cerebellum, the GFAP-IL6 mice had 369,963 ± 152,991 TSPO^+^ microglia/macrophages, which was significantly higher than that of the WT mice (12,402 ± 8820) (*p* < 0.0001) (Figures 4A,B,F). Lower doses of MC, such as 218 PPM and 436 PPM, reduced the number of TSPO^+^ microglia/macrophages by 26% (276,303 ± 40,166) and 24% (278,235 ± 35,052), respectively (Figures 4C,D). The 874 PPM MC reduced TSPO^+^ microglial/macrophages number by 42% (215,912 ± 80,444) compared with the GFAP-IL6 normal food fed mice (Figures 4E,F).

### MC Decreased GFAP^+^ Astrocytes in the Hippocampus and the Cerebellum of the GFAP-IL-6 Mice

To quantify the number of astrocytes, GFAP immunostaining and stereological quantification were performed to investigate the genotype difference between the WT and GFAP-IL6 mice. The effect of MC on the elevated number of GFAP^+^ astrocytes in the GFAP-IL6 mice was investigated in the hippocampus and cerebellum.

**FIGURE 5:**
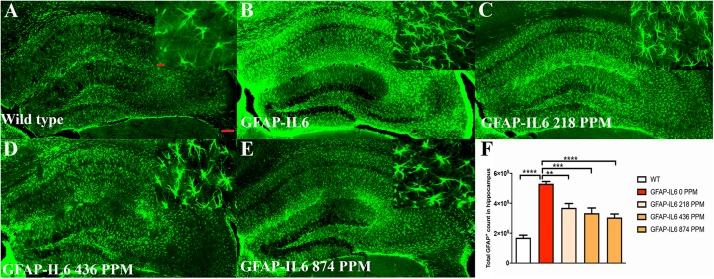
MC decreased GFAP^+^ astrocytes in the hippocampus of GFAP-IL-6 mice. **(A)** Representative photomicrographs of immunofluorescence staining for the astrocytes (GFAP^+^ cells) in the hippocampus. (Magnification 5x and 63x objective field, scale bar = 100 μm in 5x and 50 μm in 63x). Images are representative of at least six animals per group (*n* = 6). **(B–F)** Graphical representation of total GFAP count in the hippocampus. The graph represents the mean ± SEM and significant differences were determined using one-way analysis of variance (ANOVA). Significance = *p < 0.05, **p < 0.001, ***p < 0.0001, ****p < 0.0001.

In the hippocampus, the GFAP-IL6 mice had a significantly larger number of GFAP^+^ astrocytes (530,374 ± 30,447) compared with the WT mice (169,908 ± 17,046) (*p* < 0.0001) (Figures 5A,B,F). In the MC fed GFAP-IL6 mice, a significant reduction in the number of GFAP^+^ astrocytes was observed in a dose-dependent manner compared with the GFAP-IL6 normal fed group. MC at a dose of 218 PPM and 436 PPM reduced the number by 30% (369,401 ± 64,227) and 37% (333,646 ± 85,311), respectively (Figures 5C,D,F). The 874 PPM downregulated the total GFAP^+^ astrocytes by 42% (304,668 ± 62,649) (Figures 5E,F).

**FIGURE 6:**
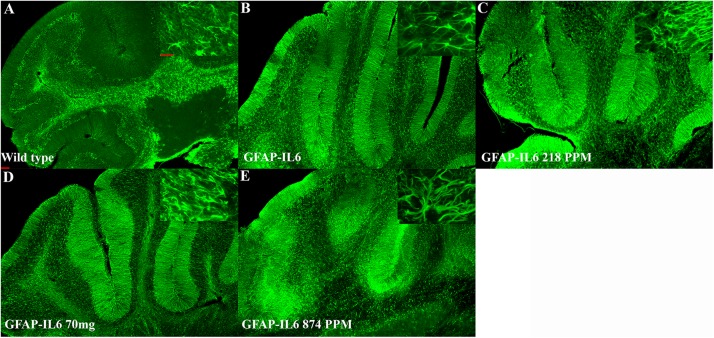
MC decreased GFAP^+^ astrocytes in the cerebellum of GFAP-IL-6 mice. Representative photomicrographs of immunofluorescence staining for the astrocytes (GFAP^+^ cells) in the cerebellum. (Magnification 5x and 63x objective field, scale bar = 100 μm in 5x and 50 μm in 63x). Images are representative of at least six animals per group (*n* = 6).

In the cerebellum, the astrocytes and their processes were very abundant and densely packed, making stereological counting difficult, hence the numbers of GFAP^+^ cells were not analyzed. However, high-level expression of GFAP protein was observed in the GFAP-IL6 mice compared with the WT mice, located mostly, but not exclusively, in the gray matter of the cerebellum, with a strong presence of elongated microglia in the white matter and molecular layer. The difference in the distribution of astrocytes between the WT and GFAP-IL6 animals was substantial. Using qualitative observation and analysis, the MC fed groups appeared to have a comparatively low expression of GFAP protein compared with the GFAP-IL6 group (Figure 6). Summary of the stereological counting is presented in Table 2.

**TABLE 2:** Stereological counting summary.

Cohort genotype	Diet	Iba-1 hippocampus (Mean ± SEM)	Iba-1 cerebellum (Mean ± SEM)	TSPO hippocampus (Mean ± SEM)	TSPO cerebellum (Mean ± SEM)	GFAP hippocampus (Mean ± SEM)
WT	Normal food	175,192 ± 12098	154,058 ± 12871	4,747 ± 1579	12,402 ± 3,601	169,908 ± 8,523
GFAP-IL6	Normal food	438,550 ± 29,717	1,025,205 ± 104,467	147,803 ± 20,895	369,963 ± 68,420	530,374 ± 15,224
GFAP-IL6	218 PPM MC	361,644 ± 20,593	693,938 ± 117,878	123,769 ± 6,266	276,303 ± 40,166	369,401 ± 28,724
GFAP-IL6	436 PPM MC	338,483 ± 33,212	592,373 ± 48,489	119,213 ± 11,229	278,235 ± 35,052	333,646 ± 34,828
GFAP-IL6	874 PPM MC	323,451 ± 21,520	605,085 ± 63,857	98,941 ± 8,094	215,912 ± 30,405	304,668 ± 23,679

### Correlation Analysis Among the Iba-1^+^ Microglia, TSPO^+^ Microglia/Macrophages, and GFAP^+^ Astrocytes

**FIGURE 7:**
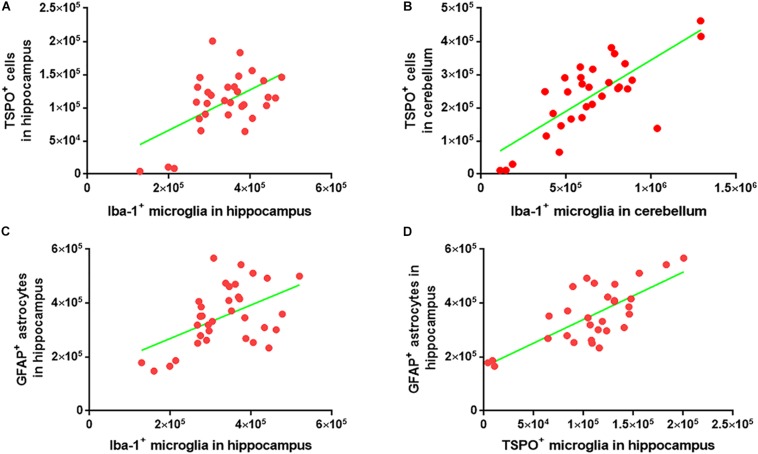
Correlation analysis among the Iba-1^+^ microglia, TSPO^+^ microglia/macrophages, and GFAP^+^ astrocytes. **(A)** Graphical representation of the correlation between Iba-1^+^ microglia and TSPO^+^ cells in hippocampus. The data were analyzed using linear regression and correlation. ^∗∗^*p* < 0.001, R2 = 0.29 (*n* = 31) **(B)** Graphical representation of the correlation between Iba-1^+^ microglia and TSPO^+^ cells in the cerebellum. The data were analyzed using linear regression and correlation. ^****^p < 0.0001, R2 = 0.58 (*n* = 31) **(C)** Graphical representation of the correlation between Iba-1^+^ microglia and GFAP^+^ astrocytes in hippocampus. The data were analyzed using linear regression and correlation. ^∗∗^*p* < 0.002, R2 = 0.25 (*n* = 34) **(D)** Graphical representation of the correlation between TSPO^+^ microglia/macrophages and GFAP^+^ astrocytes in hippocampus. The data were analyzed using linear regression and correlation. ^****^p < 0.0001, R2 = 0.54 (*n* = 30).

To investigate whether the numbers of Iba-1^+^ microglia, TSPO^+^ microglia/macrophages and GFAP^+^ astrocytes correlate within the cohorts, the Iba-1^+^ microglial count was plotted against the TSPO^+^ cell count. A strong correlation was observed between the two markers in both the cerebellum (*R* = 0.76, *p* < 0.0001) and hippocampus (*R* = 0.54, *p* < 0.0015) (Figures 7A,B). There was also a strong correlation between the Iba-1^+^ microglial number and the GFAP^+^ astrocytes number (*R* = 0.50, *p* < 0.0025) in the hippocampus (Figure 7C). Additionally, the numbers of TSPO^+^ microglia/macrophages and GFAP^+^ astrocytes were also correlated in the hippocampus (*R* = 0.54, *p* < 0.0001) (Figure 7D).

### Effect of MC on the Morphological Characteristics of Microglial Cells in the Hippocampus and Cerebellum

In order to demonstrate how chronic inflammation affects the morphology of the microglia, we analyzed the anatomical features of the Iba-1^+^ microglia in the WT and GFAP-IL6 animals in the hippocampus and cerebellum. To investigate the effects of MC on microglia morphology, the GFAP-IL6 mice fed with MC at different doses were included in the analysis.

**FIGURE 8:**
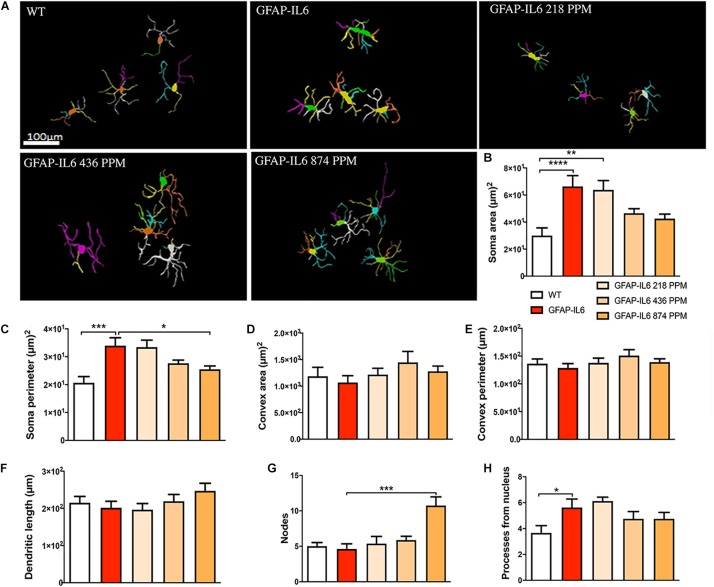
Effect of MC on the morphological characteristics of microglial cells in the hippocampus. **(A)** Morphological assessment of reactive and non-reactive microglia in the hippocampus. **(B–H)** Microglia in the inflamed mice have significantly larger soma area, soma perimeter and processes compared with the WT mice. High dose MC significantly reduced soma area and soma perimeter compared with GFAP-IL6 mice. However, the same high dose MC significantly increased the number of nodes compared with the GFAP-Il6 mice. It has no effect on the convex area, convex perimeter, dendritic length and number of processes. Significance = *p < 0.05, **p < 0.001, ***p < 0.0001, ****p < 0.0001.

In the hippocampus, a difference between the genotypes was observed, as Iba-1^+^ microglial cells of the GFAP-IL6 mice had significantly larger soma areas (66.36 ± 22.63 μm2) (*p* < 0.004), soma perimeters (33.93 ± 8.18 μm) (*p* < 0.004) and more processes (5.62 ± 1.84) (*p* < 0.05) than those of the WT mice [soma area (30.005 ± 13.93 μm2), soma perimeter (20.63 ± 5.50 μm) and number of processes (3.66 ± 1.36)]. The GFAP-IL6 mice fed with a 874 PPM dose of MC had significantly decreased soma perimeter (25.47 ± 3.44) and increased number of nodes compared with the GFAP-Il6 mice on normal food (10.75 ± 3.49) (Figure 8 and Table 3). The rest of the lower MC doses did not result in significant changes in any of the measured parameters.

**TABLE 3:** Morphological analysis of Iba-1^+^ microglia.

Cohorts	Diet	Soma area (μm)^2^ (Mean ± SEM)		Soma perimeter (μm) (Mean ± SEM)		Convex 2D (area) (μm)^2^ (Mean ± SEM)		Convex perimeter (μm) Mean ± SEM)	
		H	C	H	C	H	C	H	C
WT	Normal food	30.005 ± 5.68	30.03 ± 3.52	20.63 ± 2.24	20.55 ± 1.34	1183.93 ± 171	1197.53 ± 126.4	136.4 ± 8.50	140.93 ± 4.69
GFAP-IL6	Normal food	66.36 ± 8.0	66.64 ± 4.46	33.93 ± 2.89	33.3 ± 1.00	1067 ± 128.8	1014.54 ± 178.3	128.78 ± 7.84	121.73 ± 11.26
GFAP-IL6	218 PPM MC	63.82 ± 6.89	55.34 ± 5.30	33.43 ± 2.52	31.11 ± 2.54	1216.13 ± 119.8	1040.06 ± 33.3	137.88 ± 8.59	126.73 ± 1.87
GFAP-IL6	436 PPM MC	46.57 ± 3.31	47.62 ± 4.11	27.63 ± 1.16	26.5 ± 1.19	1446.16 ± 205.5	1570.76 ± 60.69	150.95 ± 10.72	154.92 ± 3.21
GFAP-IL6	874 PPM MC	42.55 ± 3.34	42.48 ± 4.48	25.47 ± 1.21	26.3 ± 1.60	1277.96 ± 99.78	1617.23 ± 218.1	139.21 ± 5.97	156.31 ± 12.2

**Cohorts**	**Diet**	**Total length of dendrites (μm) (Mean ± SEM)**		**Nodes** **(Mean ± SEM)**		**Dendrites** **(Mean ± SEM)**			
							
		**H**	**C**	**H**	**C**	**H**			

WT	Normal food	215.15 ± 16.99	208.81 ± 20.25	5 ± 0.51	4.33 ± 0.91	3.66 ± 0.55	4.33 ± 0.42		
GFAP-IL6	Normal food	201.55 ± 17.47	168.81 ± 24.81	4.62 ± 0.73	4.75 ± 0.67	5.62 ± 0.65	5.25 ± 0.25		
GFAP-IL6	218 PPM MC	196.47 ± 16.59	172.06 ± 14.33	5.37 ± 1.01	5.62 ± 1.14	6.12 ± 0.29	4.5 ± 0.5		
GFAP-IL6	436 PPM MC	219.16 ± 18.29	226.28 ± 9.20	5.87 ± 0.54	8.12 ± 1.21	4.75 ± 0.55	5.25 ± 0.52		
GFAP-IL6	874 PPM MC	247.0 ± 20.44	235.22 ± 23.6	10.75 ± 1.23	7.37 ± 1.08	4.75 ± 0.49	6.87 ± 0.58		

*H, hippocampus, C, cerebellum.*

**FIGURE 9:**
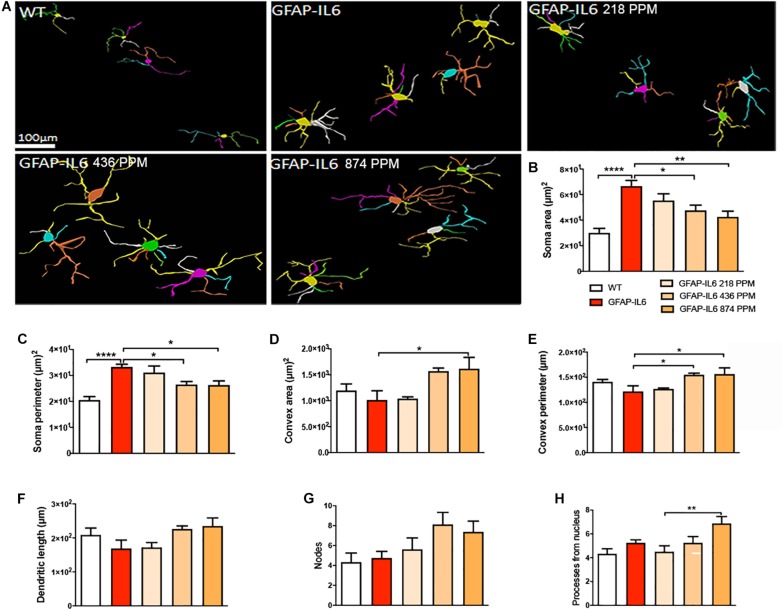
Effect of MC on the morphological characteristics of microglial cells in the cerebellum. **(A)** Morphological assessment of reactive and non-reactive microglia in the cerebellum. **(B–H)** In the cerebellum, microglia in the inflamed mice have significantly high soma area and soma perimeter compared with the WT microglia. MC significantly reduced the soma area while, MC high and medium dose with the normal curcumin significantly decreased the soma perimeter, whereas only the high dose MC significantly increase the convex area, convex perimeter, and dendritic length compared with the GFAP-IL6 mice. One-way ANOVA, Tukey’s post-test, Significance = *p < 0.05, **p < 0.001, ****p < 0.0001, mean ± SEM).

In the cerebellum, the microglial cells of the GFAP-IL6 mice fed with normal food displayed significantly larger soma areas (66.64 ± 12.62) (*p* < 0.0001) and soma perimeters (33.3 ± 2.84) (*p* < 0.0001) as compared with those of the WT mice [microglial soma area (30.03 ± 8.64) and soma perimeter (20.55 ± 0.28)]. In the MC fed mice, 436 PPM MC also reduced the soma perimeter of the microglia, while the 874 PPM dose significantly reduced the soma area (42.48 ± 12.69) (*p* < 0.0001) and soma perimeter (26.3 ± 6.52) (*p* < 0.0001), whereas it significantly increased the convex area (1617.23 ± 617.94) (*p* < 0.01), convex perimeter (156.31 ± 56.31) (*p* < 0.01), and dendritic length of the microglia (235.22 ± 35.73) (*p* < 0.05) compared with the GFAP-IL6 controls (Figure 9 and Table 3). The rest of the lower MC doses did not result in significant changes in any of the measured parameters.

**FIGURE 10:**
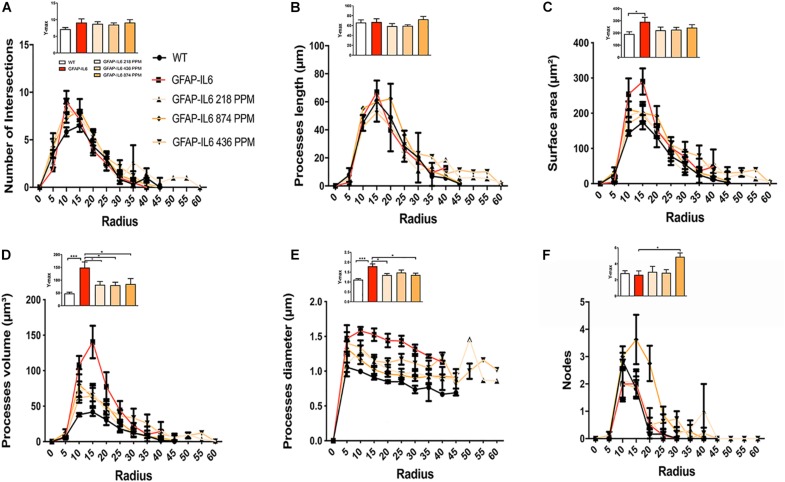
MC retracted the size of microglial cells in the hippocampus. A Sholl analysis revealed a significant change in the surface area, processes volume and processes diameter of microglia between wild type and GFAP-IL6 inflamed microglia both in the hippocampus. **(A–F)** MC retracted processes volume and processes diameter compared with the GFAP-IL6 mice. This can be observed in the peaks of the distributions of different morphological features being closer to the center compared with the WT and GFAP-IL6 mice, we call it X at Ymax. One-way ANOVA test. Significance = *p < 0.05, ***p < 0.0001.

**FIGURE 11:**
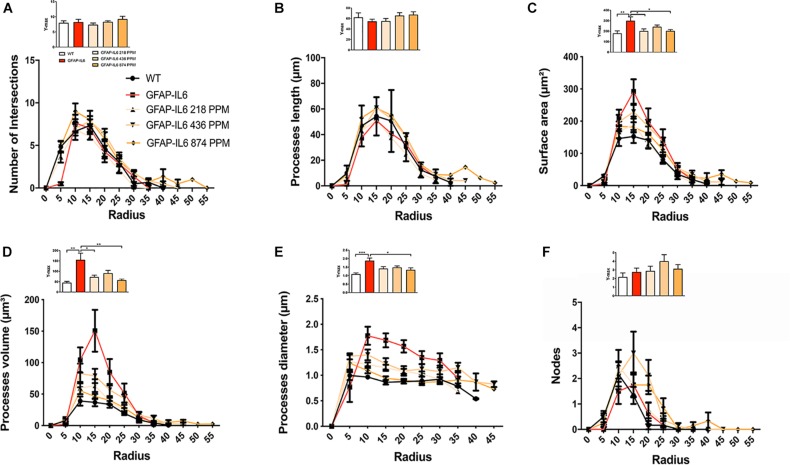
MC retracted the size if microglial cells in the cerebellum. A Sholl analysis revealed a significant change in the surface area, processes volume and processes diameter of microglia between wild type and GFAP-IL6 inflamed microglia both in the cerebellum. **(A–F)** MC retracted surface area, processes volume and processes diameter compared to the inflamed microglia. This can be observed in the peaks of the distributions of different morphological features being closer to the center compared with the WT and GFAP-IL6 mice, we call it X at Ymax. One-way ANOVA test.

Sholl analysis, a quantitative analysis to investigate the morphological characteristics, of the microglial cells from the hippocampus and the cerebellum revealed some further variations in morphology between the WT and GFAP-IL6 normal food fed, GFAP-IL6 MC fed mice characteristics. In the hippocampus, the microglia of the GFAP-IL6 mice have a significantly larger surface area (*p* < 0.03), process volume (*p* < 0.0006), and process diameter (*p* < 0.0004) than that of WT mice. In the MC fed mice, the 218 PPM and 436 PPM dose significantly reduced the process volume and diameter, whereas the 874 PPM dose significantly decreased the process volume (*p* < 0.0006) and surface area (*p* < 0.03), while it significantly increased the number of nodes (*p* < 0.02) compared with the microglia of normal fed GFAP-IL6 mice (Figure 10). In the cerebellum of the GFAP-IL6 mice, the Iba-1^+^ microglia have a significantly smaller surface area (*p* < 0.002), process volume (0.0008), and process diameter (0.001) compared with microglia of the WT mice. In the MC fed mice, the 218 PPM dose significantly downregulated the surface area (*p* < 0.02) and process volume (*p* < 0.01), while the 874 PPM dose significantly reduced the surface area (*p* < 0.002), process volume (*p* < 0.0008), and the process diameter (*p* < 0.0013) of the microglia compared with the microglia of non-fed GFAP-IL6 mice (*p* < 0.01) (Figure 11).

**FIGURE 12:**
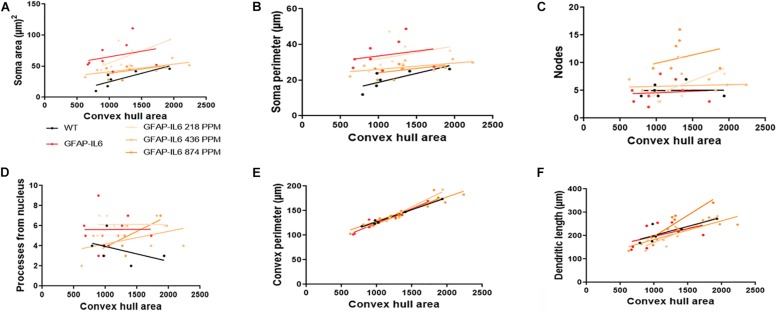
Bivariate correlation: MC effects on morphology are dose-independent in the hippocampus. **(A–F)** Different morphological parameters have been plotted against convex hull area to see the bivariate correlation between WT, GFAP-IL6 and between non-treated and treated ones. We found that the convex perimeter of both wild type and GFAP-IL6 are significantly correlated, whereas treatment with any dose of MC showed significant correlation of convex perimeter and dendritic length relative to convex hull area in the hippocampus. ANCOVA and sum-of-squares tests.

**FIGURE 13:**
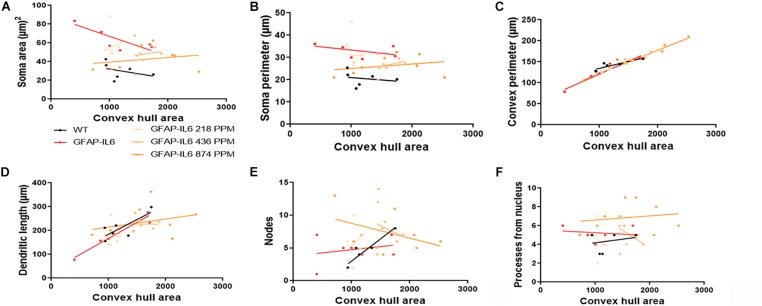
Bivariate correlation: MC effects on morphology are dose-independent in the cerebellum. **(A–F)** Different morphological parameters have been plotted against convex hull area to see the bivariate correlation between the WT, GFAP-IL6 and between non-treated and treated ones. We found that medium and a high dose of MC showed a significant correlation in convex perimeter relative to convex hull area in cerebellum ANCOVA and sum-of-squares tests.

In the hippocampus, correlation analysis of microglial cell size with each morphological characteristic revealed significant correlations with soma area in the WT and 436 PPM dose, with convex perimeter in all cohorts, and with dendritic length in the curcumin-treated cohorts, which revealed that microglial cells of the WT and 436 PPM fed groups tended to increase the size of the soma area. It also suggests that the convex perimeter of the microglia of each cohort increased with the increasing size of the cells, whereas the dendritic lengths of the microglia of the fed cohorts were increased with the overall cell size. Despite these changes, soma area, soma perimeter, nodes and number of dendrites of microglia of all treated cohorts remained consistent regardless of the cell size (Figure 12 and Table 4). In the cerebellum, some significant correlations were observed between the soma area and the dendritic length of the GFAP-IL6 microglia, whereas additional significant correlations were observed in the convex perimeter in each cohort, showing that the soma area and dendritic length of microglia change with the overall cell size, while the convex perimeter increases with the overall increase in microglial cells. The remaining perimeter measures had no effect and remained consistent regardless of the cell size (Figure 13 and Table 5).

**TABLE 4:** Bivariate correlation of morphological characteristics with overall microglial cell size in the hippocampus.

		Soma area		Soma perimeter		Convex perimeter		Dendritic length		Nodes		Primary dendrites	
Cohort	Diet	*R*^2^	Correlation	*R*^2^	Correlation	*R*^2^	Correlation	*R*^2^	Correlation	*R*^2^	Correlation	*R*^2^	Correlation
WT	Normal food	0.67	*0.0453	0.58	0.0756	0.98	**** < 0.0001	0.61	0.0665	0	0.9718	0.2	0.3629
GFAP-IL6	Normal food	0.08	0.4881	0.05	0.5626	0.92	***0.0001	0.23	0.2200	0	0.8238	0	0.9851
GFAP-IL6	218 PPM MC	0.47	0.0586	0.12	0.3955	0.95	**** < 0.0001	0.72	**0.0075	0.35	0.1211	0	0.9949
GFAP-IL6	436 PPM MC	0.58	*0.0281	0.38	0.1032	0.87	***0.0006	0.8	**0.0023	0.01	0.7821	0.21	0.2502
GFAP-IL6	874 PPM MC	0.16	0.3164	0.16	0.3178	0.95	**** < 0.0001	0.78	**0.0035	0.05	0.5621	0.4	0.0905

**TABLE 5:** Bivariate correlation of morphological characteristics with overall microglial cell size in cerebellum.

		Soma area		Soma perimeter		Convex perimeter		Dendritic length		Nodes		Primary dendrites	
Cohorts	Diet	*R*^2^	Correlation	*R*^2^	Correlation	*R*^2^	Correlation	*R*^2^	Correlation	*R*^2^	Correlation	*R*^2^	Correlation
WT	Normal food	0.13	0.4787	0.04	0.6975	0.77	*0.0204	0.59	0.0709	0.88	**0.0054	0.05	0.6602
GFAP-IL6	Normal food	0.72	**0.0076	0.26	0.1937	0.95	**** < 0.0001	0.94	**** < 0.0001	0.06	0.5462	0.04	0.6103
GFAP-IL6	218 PPM MC	0.04	0.6193	0	0.9659	0.46	0.0636	0	0.835	0.06	0.5371	0.08	0.4734
GFAP-IL6	436 PPM MC	0.02	0.7305	0.08	0.471	0.78	**0.0035	0	0.9741	0.04	0.6067	0.17	0.2969
GFAP-IL6	874 PPM MC	0.05	0.5692	0.07	0.5112	0.97	**** < 0.0001	0.09	0.4469	0.2	0.2572	0.02	0.6960

### Effect of MC on the Morphological Characteristics of Astrocytes in the Hippocampus

**FIGURE 14:**
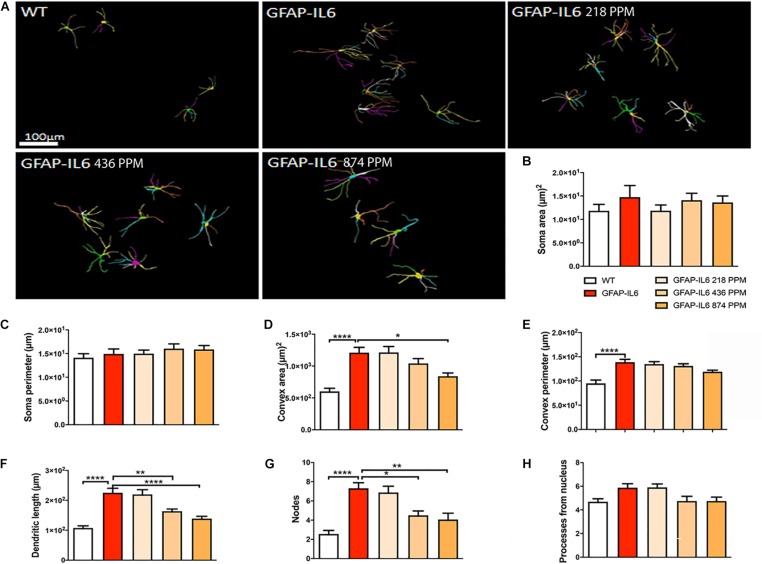
Effect of MC on the morphological characteristics of astrocytes in the hippocampus. **(A)** 3D reconstruction of astrocytes in WT and GFAP-IL6 in both treated and non-treated mice. **(B–H)** Astrocytes in the inflamed mice have a significantly high convex area, convex perimeter, dendritic length and nodes compared with the WT. High dose MC significantly decreased the convex area, dendritic length, and nodes, whereas the medium dose also significantly decreased both the dendritic length and number of nodes compared with the GFAP-IL6. One-way ANOVA, Tukey’s post-test, Significance = *p < 0.05, **p < 0.001, ****p < 0.0001 (mean ± SEM).

During inflammation, astrocytes are thought to undergo cellular hypertrophy and increased the thickness of their main cellular processes. In order to investigate the difference in morphology of the astrocytes between the WT and GFAP-IL6 normal food fed mice, the GFAP-IL6 normal food and the different doses of MC food fed mice, the morphology of astrocytes was characterized in the hippocampus. Figures 14A–F demonstrates the representative images of astrocytes immunostained for GFAP in the hippocampus. The astrocytes in the GFAP-IL6 normal food fed mice had significantly a larger convex area (1247.74 ± 371.77 μm2) (*p* < 0.0001), convex perimeter (142.27 ± 26.27 μm) (*p* < 0.0001), dendritic length (224.35 ± 66.24 μm) (*p* < 0.0001), and number of nodes (7.33 ± 2.49) (*p* < 0.0001), when compared with astrocytes of the WT mice [convex area (601.93 ± 201.46 μm2), convex perimeter (95.04 ± 27.79 μm), dendritic length (107.68 ± 29.17 μm) and number of nodes (4.68 ± 1.0). In contrast, the 436 PPM dose significantly decreased the dendritic length (160.85 ± 30.96 μm) (*p* < 0.002) and the number of nodes (4.46 ± 1.94) (*p* < 0.02), whereas the 874 PPM fed MC significantly decreased the convex area (857.27 ± 200.79 μm2) (*p* < 0.02), dendritic length (139.72 ± 32.87 μm) (*p* < 0.002), and the number of nodes (3.86 ± 2.61) (*p* < 0.02) (Table 6).

**TABLE 6:** Morphological analysis of GFAP^+^ astrocytes in the hippocampus.

Cohorts	Diet	Soma area (μm)^2^ (Mean ± SEM)	Soma perimeter (μm) (Mean ± SEM)	Convex 2D (area) (μm)^2^ (Mean ± SEM)	Convex perimeter (μm) Mean ± SEM)	Total length (μm) (Mean ± SEM)	Nodes (Mean ± SEM)	Dendrites (Mean ± SEM)
WT	Normal food	16.84 ± 1.36	14.13 ± 0.84	601.93 ± 50.37	95.04 ± 6.94	107.68 ± 7.29	2.56 ± 0.36	4.68 ± 0.25
GFAP-IL6	Normal food	13.96 ± 2.48	13.93 ± 1.04	1247.74 ± 82.95	142.27 ± 5.88	224.35 ± 14.95	7.33 ± 0.58	6.08 ± 0.34
GFAP-IL6	218 PPM MC	11.20 ± 1.23	13.86 ± 0.76	1275.35 ± 93.23	135.63 ± 5.04	249.12 ± 15.79	8.33 ± 0.65	5.77 ± 0.29
GFAP-IL6	436 PPM MC	18.67 ± 1.48	18.10 ± 0.99	1064.34 ± 75.74	131.48 ± 4.52	160.85 ± 7.52	4.46 ± 0.45	4.69 ± 0.40
GFAP-IL6	874 PPM MC	14.16 ± 1.39	16.34 ± 0.82	857.27 ± 51.17	120.36 ± 3.20	139.72 ± 7.98	3.86 ± 0.66	4.8 ± 0.33

**FIGURE 15:**
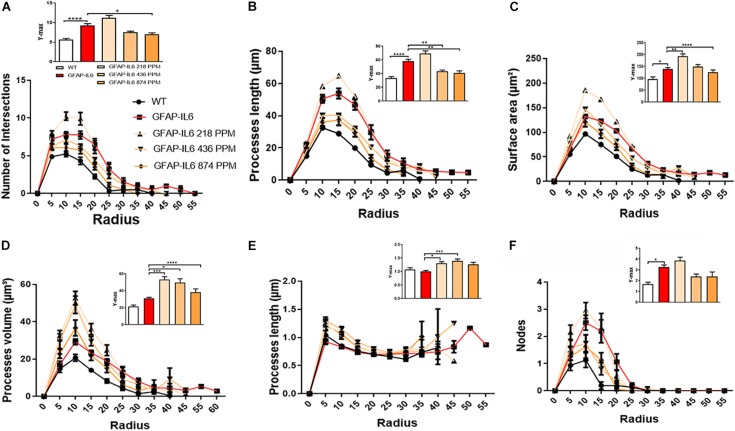
MC increased the ramification of astrocytes in the hippocampus region. **(A–F)** A Sholl analysis confirmed a significant increase in the number of the intersection, processes length, surface area, and a number of nodes compared with the WT. In contrast, MC significantly affects the inflamed astrocytes, which is observed in the peaks of the distributions of different morphological features being closer to the center compared with the WT and GFAP-IL6 mice, we call it X at Ymax. One-way ANOVA test. Significance = *p < 0.05, **p < 0.001, ***p < 0.0001, ****p < 0.0001.

Furthermore, the morphology of astrocytes was quantified by Sholl analyses. As expected from the data presented, this analysis revealed some phenotype differences in the number of intersections, processes lengths, surface areas, and a number of nodes, which were significantly larger in the GFAP-IL6 mice compared with the WT mice. In addition, it was found that both the 436 PPM and 874 PPM MC doses significantly decreased the process length. Interestingly, the 218 PPM MC dose increased the surface area, while all doses resulted in increased process volume and length of the processes compared with the non-fed GFAP-IL6 mice. No effects of MC were observed in the number of nodes (Figure 15).

**FIGURE 16:**
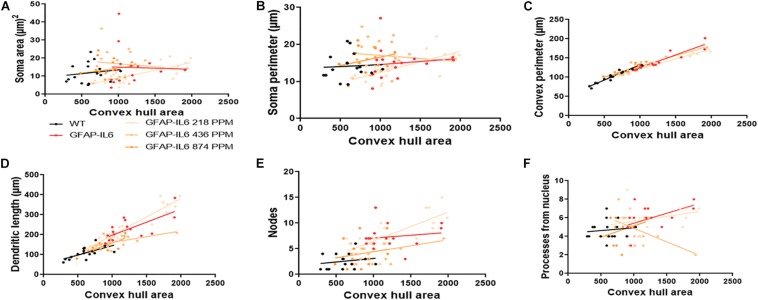
Bivariate correlation: MC effects on astrocytes morphology are dose independent in the hippocampus. **(A–F)** Different morphological parameters have been plotted against convex hull area to see the bivariate correlation between the WT, GFAP-IL6 and between nontreated and treated astrocytes. The analysis revealed that the convex perimeter and dendritic length are significantly correlated irrespective of the MC dose, whereas in the case of a low dose of MC, soma area, soma perimeter and nodes while the primary dendrites in medium dose, are significantly correlated relative to convex hull area. Analyzed by ANCOVA and sum-of-square tests.

The bivariate correlations of morphological characteristics were performed with overall astroglial cell size to investigate the impact of the size of the astroglial cells on the structural parameters. Some positive correlations of the astroglial cell size with morphological parameters were observed. Soma area and soma perimeter of astrocytes in the 218 PPM MC group changed significantly with the overall change in the entire cells. The convex perimeter and dendritic length of all cohorts changed with the overall changes in astrocytes. It was also observed that the number of nodes in the astrocytes of the 218 PPM and 874 PPM fed cohorts decreased when the overall size of the astrocytes decreased. In the case of primary dendrites, both the 436 PPM and 874 PPM doses positively decreased them with the decrease in the overall cell size of astrocytes (Figure 16 and Table 7). Taken together, the morphological analyses of the study have revealed that the highest MC dose was able to decrease astrocyte activation and reduced its size similar to that of the astrocytes in the WT mouse.

**TABLE 7:** Bivariate correlation of morphological characteristics with overall astroglial cell size in the hippocampus.

		Soma area		Soma perimeter		Convex perimeter		Dendritic length		Nodes		Primary dendrites	
Cohorts	Diet	R^2^	Correlation	R^2^	Correlation	R^2^	Correlation	R^2^	Correlation	R^2^	Correlation	R^2^	Correlation
WT	Normal food	0.02	0.5654	0	0.8276	0.93	**** < 0.0001	0.42	**0.0064	0.03	0.4794	0.01	0.6605
GFAP-IL6	Normal food	0	0.8855	0.01	0.63	0.88	**** < 0.0001	0.52	**0.0014	0.02	0.561	0.27	*0.0375
GFAP-IL6	218 PPM MC	0.56	***0.0001	0.48	***0.0006	0.97	**** < 0.0001	0.88	**** < 0.0001	0.53	***0.0003	0.11	0.139
GFAP-IL6	436 PPM MC	0.01	0.6311	0.01	0.6725	0.82	**** < 0.0001	0.33	*0.018	0.15	0.1357	0.3	*0.0272
GFAP-IL6	874 PPM MC	0.04	0.4396	0.07	0.2938	0.79	**** < 0.0001	0.65	***0.0001	0.03	0.4876	0.13	0.1603

## Discussion

Chronic neuroinflammation is considered to be one of the major contributing factors in the progression of various neurodegenerative diseases, such as dementia, Alzheimer’s and Parkinson’s disease (Van Eldik et al., 2007). This study suggests that the cytokine-suppressive anti-inflammatory drug curcumin is able to downregulate chronic glial activation and protect against inflammation-induced neuronal damage, as we have previously suggested (Venigalla et al., 2016). Curcumin, a common spice, has been used for many decades as a drug for numerous inflammatory disorders. Despite its various pharmacological and therapeutic activities, curcumin’s poor absorption, low permeability, and low bioavailability have limited its therapeutic effects. To overcome these limitations, a number of different formulations have been created to increase its absorption or inhibit its metabolization, such as the addition of piperine, formulation of liposomal curcumin, curcumin nanocapsules and phospholipid curcumin complexes (Anand et al., 2007; Tsai et al., 2011). Studies have reported that curcumin can exert a broad range of anti-inflammatory effects in the CNS (Abe et al., 1999; Drion et al., 2018). Curcumin preparations, such as Longvida^®^ (VS Corp), have achieved higher concentrations in the brains of mice compared with normal curcumin, and in human subjects, led to significantly improved working memory and mood after a 4-week treatment in a randomized, double-blind, placebo-controlled trial (Begum et al., 2008; Wu et al., 2014). In this study, MC was fed to the GFAP-IL6 mice to investigate the potential therapeutic effects of MC in chronic neuroinflammation. In this model, the murine IL-6 gene is expressed in astroglia under the transcriptional control of the murine glial fibrillary acidic protein (GFAP) promoter, resulting in cytokine-induced low-grade chronic neuroinflammation (Campbell et al., 1993) presenting with microglial activation and increased levels of pro-inflammatory markers throughout the life span (Gyengesi et al., 2019). Using this model, the anti-inflammatory effects of three doses of MC were investigated compared with the normal fed GFAP-IL6 mice.

After feeding the mice with MC for 4 weeks, it was observed that MC reduced the number of Iba-1^+^ microglia in the cerebellum and the hippocampus, with the high dose of MC resulting in the most substantial decrease. Previous studies have reported that the numbers of Iba-1^+^ microglia and GFAP^+^ astrocytes in the mouse brain increase in chronic neuroinflammatory conditions, which were significantly reduced after being treated with anti-inflammatory drugs such as Tenilsetam (Gyengesi et al., 2018). A recent study conducted in 5 x FAD mice, a mouse model of familial Alzheimer’s disease, has reported the protective effect of a solid lipid curcumin formulation (Maiti et al., 2018). They used Image-J software to count the number of GFAP^+^ and Iba-1^+^ cells and reported that the curcumin formulation was able to significantly downregulate the microglial and astroglial numbers in different regions of the hippocampus in the mice brains (Maiti et al., 2018). Furthermore, they reported that their formulation of curcumin has higher permeability into brain tissue and superior therapeutic effects in relation to the reduction of amyloid plaque formation relative to normal curcumin (Maiti et al., 2018).

Our study found that the total estimated number of TSPO^+^ cells in the cerebellum and the hippocampus was significantly larger in the GFAP-IL6 group compared with the WT group. The 874 PPM dose of MC significantly reduced TSPO^+^ cell number in the GFAP-IL6 mice in both the hippocampus and the cerebellum compared with the non-fed group. A study conducted in mice that measured the fluorescence intensity and the number of TSPO^+^ cells supports our findings, wherein the number of Iba-1^+^ and TSPO^+^ microglia/macrophages in the GFAP-IL6 mice was significantly larger compared with the WT mice (Bonsack et al., 2016).

Additionally, all three doses of MC significantly decreased the number of reactive astrocytes in the hippocampus compared with the GFAP-IL6 control mice in a dose-dependent manner.

Within our cohorts, WT, GFAP-IL6, and GFAP-IL6 MC treated microglial morphologies were characterized in GFAP-IL6 mice, which revealed certain characteristics of microglia not reported elsewhere. The present study has identified that the microglial soma area and soma perimeter significantly increased in both the hippocampus and cerebellum of the GFAP-IL6 mice compared with those of the WT’s. Additionally, there was a significantly larger number of processes in the microglia of the inflamed brains compared with the non-inflamed brains. MC altered the morphology of the microglial cells. The high dose MC significantly reduced the soma area and soma perimeter, while it significantly increased the number of nodes in the hippocampus. In the cerebellum, the high dose MC significantly reduced the soma area and soma perimeter, while it increased the convex area, convex perimeter, and dendritic length compared with the non-fed control. In short, the MC modified morphology toward that of the microglia in the WT mice.

Ramified microglia function in a highly dynamic manner, constantly surveying the neuronal environment for homeostatic and infective changes and protecting hippocampal neurons under pathological conditions (Vinet et al., 2012). It has been reported that microglial cells are activated in response to many types of neuronal injury or inflammation, and undergo morphological transformations (Nimmerjahn et al., 2005). Moreover, resting microglia have the ability to facilitate prompt reactions to brain injury (Nolte et al., 1996). Studies have shown that changes in microglial cell size can influence the entire morphology in response to different challenges to the CNS (Hinwood et al., 2013). A recent study conducted in mice has shown that curcumin can reduce the aggregation and ramification of microglial cells in the hippocampus. They reported that the microglia were more aggregated in the vehicle-treated group, while, after injecting solid lipid curcumin to the mice for 5 days, they noticed a significant reduction in microglial aggregation, activation, and branch number (Maiti et al., 2018). Herein, morphological investigations of astrocytes in the hippocampus have shown that astrocytes of GFAP-IL6 mice have significantly longer dendrites compared with those of the WT mice, and the high and medium dose of MC significantly decreased the dendritic length. Moreover, the convex area of the inflamed astrocytes was also significantly larger than that of the WT-type mice cells, with the high dose MC significantly decreasing the convex area. Emerging studies have reported that curcumin can inhibit the hypertrophy of astrocytes in the CNS. One such study has reported that a curcumin formulation was able to decrease astrocyte activation and the number of branches of the astrocytes in the rat hippocampus (Cerbai et al., 2012). Similarly, another study conducted in a rat injury model has reported a decrease in hypertrophy of astrocytes after been treated with curcumin (Ji et al., 2013; Maiti et al., 2018).

One of the limitations of the study, however, is that the heating and melting of phytosomal curcumin during while in contact with powdered chow could potentially destroy the phytosome when the liquified phospholipids can interact with chow particles.

Altogether, our study has demonstrated that MC is able to downregulate chronic neuroinflammation in the GFAP-IL6 mouse brain measured by various markers of neuroinflammation.

## Data Availability Statement

All data and materials described in the article, including all relevant raw data, will be freely available to any scientists upon request from the corresponding author. Minimal (averaged from raw data) data sets are included within the article as Tables 2–7.

## Ethics Statement

The experimental procedures were approved by the Western Sydney University Animal Care and Ethics Committee (Approval ID: A11393) and carried out in accordance with the rules established by the National Health and Medical Research Council of Australia.

## Author Contributions

GN bred and genotyped the animals and contributed to the manuscript drafting and editing. FU and HL collected the tissue, performed the histological staining, analyzed the data, and prepared the draft of the manuscript. GM conceptualized the study, supervised the data collection, and edited the manuscript. EG designed and supervised the experiments, edited and corrected analysis and the manuscript. All authors have reviewed the manuscript.

## Conflict of Interest

The authors declare that the research was conducted in the absence of any commercial or financial relationships that could be construed as a potential conflict of interest. EG and GM discloses a collaboration with Indena S.p.A, Italy, which had no role in the conceptualization and writing of the article, and in the decision to submit the paper for publication.
