# Supplementary material for: Evaluation of Phytosomal Curcumin as an Anti-inflammatory Agent for Chronic Glial Activation in the GFAP-IL6 Mouse Model
Source: Front Neurosci. 2020 Mar 12;14:170. doi: 10.3389/fnins.2020.00170 (PMC7081170; doi:10.3389/fnins.2020.00170)
Supplement: Supplementary file 1 [file Table_1.docx]

**Evaluation of Meriva curcumin phytosome as an anti-inflammatory agent for chronic glial activation in the GFAP-IL6 mouse model**

**Faheem Ullah^1^, Huazheng Liang^1, 2^, Garry Niedermayer^3^, Gerald Münch^1,4^, Erika Gyengesi^1*^**

1. Department of Pharmacology, School of Medicine, Western Sydney University, Campbelltown, NSW, Australia
2. Department of Neurology, Shanghai Fourth People’s Hospital, Tongji University, Shanghai, China

3. School of Science, Western Sydney University, Campbelltown, NSW, Australia

4. NICM Health Research Institute, Western Sydney University, Campbelltown, NSW, Australia

***Supplementary table 1.***

| **Intended dose (mg/ kg/bw/day)** | 140 | 70 | 35 |
| --- | --- | --- | --- |
| **MC preparation content in chow (g/kg)** | 4.37 | 2.18 | 1.09 |
| **Curcumin content in chow (g/kg)** | 0.874 | 0.436 | 0.218 |
| **Curcumin content in chow (ppm)** | 874 | 436 | 218 |
| **Average daily food consumed (g)** | 3.329 | 3.29 | 3.76 |
| **Curcumin consumed (mg/day)** | 2.87546 | 1.43444 | 0.81968 |
| **Average body weight (g)** | 25.9 | 25.74 | 23.75 |
| **Curcumin consumed per bodyweight (mg/g)** | 0.0345 | 0.0557 | 0.1110 |
| **Actual curcumin dose consumed (mg/ kg/bw/day)** | 111.02 | 55.72 | 34.512 |
| **Table 1.** ***Meriva curcumin daily dose calculation***  The actual average daily curcumin dosing of the mice (mg/kg bodyweight per day as well as PPM) calculated. The average bodyweights of the mice and their daily Meriva curcumin food consumption were calculated by using their average bodyweight and an average food consumption values measured weekly between the beginning and the end of the feeding period (4 weeks). | | | |
